# CYP2D6 genotype and outcome in tamoxifen treated early breast cancer

**DOI:** 10.2340/1651-226X.2025.43208

**Published:** 2025-07-02

**Authors:** Linda Thorén, Jonatan D. Lindh, Espen Molden, Marianne Kristiansen Kringen, Jonas Bergh, Erik Eliasson, Sara Margolin

**Affiliations:** a Department of Clinical Science and Education at Södersjukhuset, Karolinska Institutet, Stockholm, Sweden; b Department of Oncology, Södersjukhuset, Stockholm, Sweden; c Department of Laboratory Medicine, Division of Clinical Pharmacology, Karolinska Institutet, Stockholm, Sweden; d Karolinska University Hospital, Medical Diagnostics Function, Clinical Pharmacology, Stockholm, Sweden; e Department of Pharmaceutical Biosciences, School of Pharmacy, University of Oslo, Oslo, Norway; f Center for Psychopharmacology, Diakonhjemmet Hospital, Oslo Norway; g Department of Life Sciences and Health, Oslo Metropolitan University, Oslo, Norway; h Department of Oncology-Pathology, and Breast Cancer Center Karolinska Comprehensive Cancer Center, Karolinska Institutet, Karolinska University Hospital, Stockholm, Sweden

**Keywords:** CYP2D6, tamoxifen, breast cancer, adjuvant, bioactivation, menopausal status, pharmacogenetics

## Abstract

**Background and purpose:**

The clinical significance of individual CYP2D6 activity for the outcome of tamoxifen treatment in early breast cancer is unclear. Our previous investigation in patients diagnosed over the period 1998–2000 indicated an association between reduced CYP2D6 activity and poor outcome in premenopausal women. The aim of this study was to investigate the association between CYP2D6 genotype and clinical outcome in a larger tamoxifen treated cohort.

**Patients/material and methods:**

Swedish breast cancer patients who initiated adjuvant tamoxifen treatment over the period 2006–2014 constituted the full study cohort. Clinical information was collected from medical records. Data on endocrine treatment, use of CYP2D6 inhibitors was retrieved from the Swedish Prescribed Drug Register. *CYP2D6* was genotyped and translated into predicted metabolic activity. The association between CYP2D6 activity and clinical outcome was analyzed using Cox regression, controlling for potential confounding variables. Subgroup analyses were performed based on menopausal status, tamoxifen treatment for at least 1 year and as single endocrine treatment, HER2-status and tamoxifen monotherapy.

**Results:**

A total of 1,103 patients were included. A total of 761 patients received tamoxifen as monotherapy. A total of 42% were premenopausal. Median follow-up was 11.4 years. No significant association was found between CYP2D6 activity and recurrence (adjusted hazard ratio [aHR] 1.18, 95% CI 0.92; 1.52) or breast cancer mortality (aHR 1.41, 95%CI 0.93; 2.13) in the full cohort, or in the subgroup with tamoxifen monotherapy (aHR 1.39, CI 0.99; 1.96 and 1.88, CI 0.98; 3.60 respectively).

**Interpretation:**

No association was noted between reduced CYP2D6 activity and poorer outcome in this early breast cancer cohort, with patients generally at lower risk of recurrence, reflecting the role of adjuvant tamoxifen in current clinical practice.

## Introduction

Adjuvant treatment with 5 years of tamoxifen reduces the risk for relapse substantially not only during treatment, but also during the subsequent decade in patients with estrogen receptor (ER) positive breast cancer [1]. For breast cancer recurrence within 15 years, the relative risk reduction is nearly 40% while the absolute risk reduction is 13% [1]. The corresponding relative and absolute reduction in breast cancer mortality is 30 and 9% respectively [1]. Extended tamoxifen treatment for 10 years improves outcome further, [2]. There is however a wide inter-patient variability in the clinical outcome of tamoxifen treatment, including tolerability issues [3, 4], so further personalization of the treatment is clinically important.

Inter-individual variability in the metabolic conversion of tamoxifen to its active metabolite endoxifen has been linked to genetic polymorphism of the *CYP2D6* gene [5]. In patients carrying genotypes encoding no CYP2D6 activity, that is poor metabolizers (PM) comprising 5–10% of Caucasian European populations, the treatment effect of adjuvant tamoxifen has been reported to be reduced [6–8].

Our previous study in a cohort of 382 tamoxifen treated patients, diagnosed between 1998 and 2000, indicated an association between CYP2D6 activity and outcome mainly in premenopausal patients [9]. Our findings were later supported by similar results in a premenopausal cohort by Saladores and colleagues [10]. However, due to conflicting findings reported by other investigators [11–15], *CYP2D6* genotyping has not yet been implemented in clinical practice to guide tamoxifen treatment. More knowledge is therefore needed to better understand the clinical relevance of genetic variability in CYP2D6 metabolism for clinical outcomes in early breast cancer patients subject to tamoxifen treatment, especially in a modern clinical setting with patients receiving multimodal adjuvant therapy.

Suboptimal adherence to tamoxifen, with a negative influence on outcome [16–19] is an important clinical problem. Genetic polymorphism in CYP2D6 has been reported to influence adherence to tamoxifen [4]. Moreover, concomitant medication with potent CYP2D6 inhibitors may potentially reduce bio-activation of tamoxifen [20, 21].

The primary aim of the present study was to further investigate the role of CYP2D6 for the outcome in a larger cohort of tamoxifen-treated early breast cancer, accounting for adherence to tamoxifen and exposure to potent CYP2D6 inhibitors.

## Patients/materials

**Figure 1:**
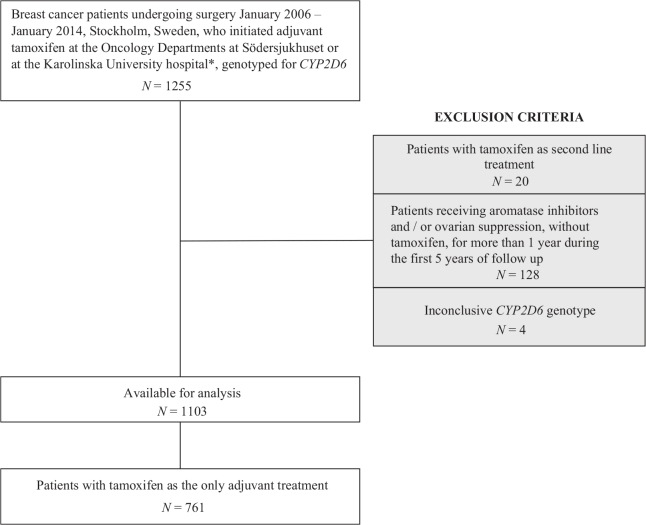
Flow chart of study participant selection and reasons for exclusion. *When the study patients were included, the Oncology Department was one unit, with two sites (Radiumhemmet and Södersjukhuset) and since 2016 two separate Oncology Departments; Departments of Oncology at Södersjukhuset and Breast Cancer Center, Cancer Theme at the Karolinska University Hospital.

DNA from blood has for several years been bio-banked from newly diagnosed early breast cancer patients at Södersjukhuset and the Karolinska University Hospital, Stockholm, Sweden. Using the National Quality Registry for Breast Cancer [22], we identified 1,255 patients undergoing breast cancer surgery over the period January 2006 – January 2014, who initiated adjuvant tamoxifen treatment at the Departments of Oncology, at Södersjukhuset or at the Karolinska University Hospital, with germ-line DNA available for *CYP2D6* genotyping. Subsets of the cohort have been analyzed in previous studies [23–25]. Patients were excluded if they initiated treatment with an aromatase inhibitor (AI) or ovarian suppression alone rather than tamoxifen as their first adjuvant endocrine therapy, or if their *CYP2D6* genotype was inconclusive. To minimize a significant effect of other endocrine treatments on the outcome [26], patients receiving AIs and/or ovarian suppression, without tamoxifen, for more than 1 year during the first 5 years of follow-up were excluded. Patients were monitored from the date of their first tamoxifen dispensation, until local breast cancer recurrence, distant metastasis, contralateral cancer, death or end of follow-up. A detailed description of the selection of the study population is depicted in Figure 1.

Data on menopausal status at breast cancer diagnosis, tumor characteristics, breast cancer treatment and follow-up were retrospectively obtained from medical records. To obtain as correct information as possible on adherence to tamoxifen, other endocrine treatment and possible concomitant treatment with CYP2D6 inhibitors, data from all filled prescriptions between January 2006 and January 2018 on adjuvant endocrine therapy and clinically relevant CYP2D6 inhibitors were retrieved from the National Prescribed Drug Register, covering all prescribed drugs in Sweden [27], as described previously [25].

### Genotyping of CYP2D6

The bio-banked DNA (retrieved from whole blood) was stored at –80ºC and transported frozen for analysis. Analysis of CYP2D6 variant alleles was performed by TaqMan-based real time polymerase chain reaction assays, validated and implemented for routine clinical pharmacogenetic analyses at Diakonhjemmet Hospital in Oslo, Norway as described previously [23]. The *CYP2D6* genotyping panel was based on established recommendations [28] and included the nonfunctional (null) variants CYP2D6*3 (rs35742686), CYP2D6*4 (rs3892097) and CYP2D6*6 (rs5030655), the decreased-function variants CYP2D6*9 (rs5030656), CYP2D6*10 (rs1065852) and CYP2D6*41 (rs28371725). Copy-number analysis (CNA) was performed to detect either one or two gene deletions (i.e. heterozygous (CN = 1) or homozygous (CN = 0) for the null-allele CYP2D6*5) or increased-function variants due to gene copies (CN = 3 or 4, instead of normally 2) [23].

### Predicted CYP2D6 activity

Each *CYP2D6* allele was given an activity score (AS). The sum of the AS values assigned to each allele was used to classify the patients into predicted CYP2D6 phenotypes; PM, (AS = 0), intermediate metabolizers (IM) (AS = 0.25 or 1.0), normal metabolizers, NM, (AS = 1.5–2.25) or ultrarapid metabolizers, UM, (AS > 2.25), in accordance with recommendations from the Clinical Pharmacogenetics Implementation Consortium and Dutch Pharmacogenetics Working Group [29]. Whilst current guidelines designate CYP2D6*41 with an AS of 0.5, data from several pharmacogenetic studies indicate a lower AS of this variant allele, that is about 0.1–0.2 [23, 30]. We therefore, in addition to the recommended AS of 0.5, analyzed the data using an AS of 0.15 for CYP2D6*41.

### Clinical outcomes

The end points of the study were breast cancer recurrence, defined as local, regional or distant recurrence, or a contralateral breast cancer, and breast cancer specific mortality.

### Statistical analysis

The association between CYP2D6 activity and breast cancer recurrence or breast cancer-related mortality was analyzed using multivariable Cox proportional hazard models. The predicted CYP2D6 activity was encoded/determined by diplotype AS as a continuous variable. The calculated hazard ratios (HRs) indicate the relative difference in hazard between a pair of individuals differing in CYP2D6 activity by a magnitude equal to a 1-unit increase in CYP2D6 activity (e.g. a CYP2D6PM with no (0) enzyme activity versus a CYP2D6IM), (1), or a CYP2D6IM, (1), versus a CYP2D6NM,(2). Supplementary categorical analyses were also performed comparing CYP2D6 PM with non-CYP2D6 PM and, using the log rank test, low CYP2D6 activity with high CYP2D6 activity (i.e. 50% enzyme activity or lower versus higher than 50% activity compared with the ‘normal’ activity encoded by CYP2D6*1/*1). Adjustments in the Cox proportional hazard models were made for the following covariates: age at time of breast cancer diagnosis, menopausal status (premenopausal yes/no, or uncertain menopausal status yes/no), medication known to inhibit CYP2D6 at any period during the first 5 years of follow-up (yes/no), having a high estimated risk of recurrence (yes/no) and adherence to tamoxifen. Adherence was defined as the proportion of the individual follow-up time (up to 5 years) that was covered by tamoxifen dispensations, that is the medication possession ratio (MPR)[31]. It was calculated as the length of follow-up in days divided by the number of tamoxifen doses dispensed during the 5-year period after tamoxifen initiation (disregarding any doses lasting beyond the end of the 5-year period). The risk of recurrence was estimated by a compilation of prognostic factors including nodal status, tumor grade, lymph node- and HER2 status and a marker of cellular proliferation (Ki-67/S phase) [32]. Ki-67 > 20% was used to differentiate between low and high values according to the definition from the St Gallen International Expert Consensus at the time for data collection [33]. Patients at high risk of recurrence were defined as having positive lymph nodes and/or tumors with a high proliferation rate (proliferation index, Ki_67_ > 20/S phase > 10%) and/or grade III and/or HER2 amplification and/or having received chemotherapy. A subgroup analysis where the main analyses were repeated separately for pre- and postmenopausal patients was also performed. Tamoxifen for 1 year is the shortest duration found to be therapeutically effective in the early clinical trials [34]. A separate analysis of all patients as well as for pre- and postmenopausal patients separately, with at least 1 year’s initial treatment on tamoxifen, not accounting for adherence thereafter, was also performed. We also performed subgroup analyses based on HER2 status, patients who did not receive any other endocrine treatment apart from tamoxifen but could have received other adjuvant systemic treatment as clinically appropriate and finally with the group of patients with tamoxifen as their only systemic postoperative therapy.

The Kaplan–Meier method was used to estimate survival. For the survival analysis patients were, as in our previous study [9], divided into two groups according to predicted CYP2D6 activity, that is 50% enzyme activity or lower versus higher than 50% activity compared with the ‘normal’ activity encoded by CYP2D6*1/*1 and for the above-described estimated groups of enzyme activity. The cutoff at 50% was selected in order to achieve approximately equally sized groups, as the groups of CYP2D6 PM and UM were small.

Time at risk was calculated from the date of tamoxifen initiation. In the analysis of time to breast cancer recurrence, data from patients without recurrent breast cancer was censored at the last follow up date and in the analysis of time to breast cancer-related death data was censored at the date of death (in patients without relapse) or on the last date in 2022 when the patients’ vital status was determined.

In all analyses, *p*-values < 0.05 (two-sided) were considered statistically significant. All statistical analyses were performed using R 4.3.1 (R Core Team (2019). R: A language and environment for statistical computing. R Foundation for Statistical Computing, Vienna, Austria. URL https://www.R-project.org/).

## Results

A total of 1,103 tamoxifen treated early breast cancer patients were included in this study. Seven per cent were defined as CYP2D6 PM, 37% as IM, 53% as NM and 3% as UM. Allele and diplotype frequencies were in Hardy–Weinberg equilibrium. Forty-two per cent were premenopausal, 39% had a high estimated risk of recurrence. Most of the patients (84%) did not receive any other adjuvant endocrine treatment apart from tamoxifen. Twenty-six per cent (*n* = 264, 57%) of the premenopausal patients and (*n* = 22, 4%) of the postmenopausal women also received chemotherapy. A total of 69% (*n* = 761) of the patients did not receive any systemic treatment other than tamoxifen. Five per cent changed their adjuvant endocrine therapy from tamoxifen to an AI, the majority due to side effects of tamoxifen. The median treatment duration for tamoxifen was 5 years. Thirty-three per cent, mainly premenopausal patients, received extended tamoxifen treatment. The median follow-up time was 11.4 years. Baseline characteristics of the included patients are summarized in Table 1.

**Table 1:** Baseline characteristics of study participants.

Baseline characteristics	Value
*N*	1,103
Age at breast cancer diagnosis, median, range (IQR)	56, 21–89 (47–66)
Premenopausal at breast cancer diagnosis, *n*	461 (41.7%)
Postmenopausal at breast cancer diagnosis, *n*	626 (56.8%)
Perimenopausal/uncertain menopausal status, *n*	16 (1.4%)
Age at menopause, median, (IQR)	50 (48–52%)
ER positive, *n*^a^	1,097 (99.5%)
ER negative, *n*^b^	3 (0.3%)
Missing, *n*	3 (0.3%)
PR positive, *n*	937 (84.9%)
PR negative, *n*	158 (14.3%)
Missing, *n*	8 (0.7%)
Tumor size,	
< 20 mm, *n*	787 (71.4%)
21–50 mm, *n*	251 (22.8%)
> 50 mm, *n*	62 (5.6%)
Missing, *n*	3 (0.3%)
Tumor grade	
I, *n*	363 (32.9%)
II, *n*	548 (49.7%)
III, *n*	173 (15.7%)
Missing, *n*	19 (1.7%)
Lymph node status	
N_0_, *n*	898 (81.4%)
N^+^, *n*	198 (17.9%)
Missing, *n* (%)	7 (0.6%)
Ki_67_ < 20/S phase < 10%, *n*	268 (24.3%)
Ki_67_ > 20/S phase > 10 %, *n*	802 (72.7%)
Missing, *n*	33 (3.0%)
HER2 positive, *n*	56 (5.1%)
Missing, *n*	15 (1.4%)
‘High risk patients’^c^, *n*	435 (39.4%)
Chemotherapy	
All patients, *n*	289 (26.2%)
Premenopausal patients, *n*^d^	264 (57.2%)
Postmenopausal patients, *n*^d^	22 (3.5%)
Uncertain/perimenopausal status, *n*^d^	3 (1.9%)
Endocrine treatment full cohort	
Tamoxifen as the single endocrine treatment, *n*	932 (84.4%)
Tamoxifen as the only systemic adjuvant treatment, *n*	761 (69%)
Tamoxifen and goserelin, *n*	93 (8.4%)
Tamoxifen and aromatase inhibitor, *n*	50 (4.5%)
Tamoxifen, goserelin and aromatase inhibitor, *n*	6 (0.5%)
Endocrine treatment premenopausal patients	
Tamoxifen as the only endocrine treatment, *n*	325 (70.4%)
Tamoxifen and goserelin, *n*	93 (20.2%)
Tamoxifen and aromatase inhibitor, *n*	21 (4.6%)
Tamoxifen, goserelin and aromatase inhibitor, *n*	6 (1.3%)
Endocrine treatment postmenopausal patients	
Tamoxifen as the only endocrine treatment, *n*	593 (94.7%)
Tamoxifen and goserelin, *n*	-
Tamoxifen and aromatase inhibitor, *n*	27 (4.3%)
Tamoxifen, goserelin and aromatase inhibitor, *n*	-

ER: estrogen receptor.

a Tumors were considered Estrogen Receptor (ER) positive and Progesterone Receptor (PR) positive if ≥ 10% of the cells stained positive for the receptor by immunohistochemistry.

b Three patients were ER-negative, but PR positive and thus defined as Hormone Receptor (HR) positive. The three patients where ER-status was missing were treated as HR-positive.

c Patients were considered at ‘high risk’ for recurrence if tumor grade III and/or Ki67 > 20/S phase > 10% and/or N^+^, and/or HER2-positive and/or treated with chemotherapy (d) Proportion of pre-, peri-, or postmenopausal patients treated with chemotherapy.

A total of 128 (12%) patients had a recurrence (whereof 63 with distant metastases) and 49 (4%) died from breast cancer during follow-up. Overall survival was 89%. A total of 73 patients were lost to follow-up during the study period and were censored at the date of their last follow-up.

In the adjusted cox regression analyses encoding the predicted CYP2D6 activity as a continuous variable, CYP2D6 activity had no statistically significant influence on either breast cancer recurrence (adjusted HR [aHR] 1.18, 95% CI 0.92; 1.52) or breast cancer mortality (aHR 1.41, CI 0.93; 2.13) in the whole study cohort of patients who initiated tamoxifen as part of their adjuvant treatment.

When separately analyzing premenopausal patients, no effect of CYP2D6 activity was found on relapse (aHR 0.99, CI 0.69; 1.42), or breast cancer specific mortality (aHR 1.14, CI 0.63; 2.05). In the postmenopausal subgroup, CYP2D6 activity had no statistically significant influence on breast cancer recurrence (aHR1.44, CI 0.99; 2.07). In this subgroup, an association between increasing CYP2D6 activity and an *increased* risk of breast cancer specific mortality was seen (aHR 1.90, CI 1.02; 3.55).

Focusing on the subgroup of patients with tamoxifen as their only systemic treatment did not reveal any statistically significant association between CYP2D6 activity and breast cancer recurrence (aHR 1.39, 95% CI 0.99; 1.96) or breast cancer mortality (aHR 1.88, CI 0.98; 3.60).

Using the alternative AS for CYP2D6*41 did not alter the findings (data not shown). Neither did stratifying for patients with tamoxifen as their only endocrine treatment, for HER2 status, nor comparing patients with low versus high CYP2D6 activity, as presented in Supplementary Tables 1 and 2. Comparing CYP2D6PM with non-CYP2D6PM did not alter the findings (data not shown).

**Figure 2:**
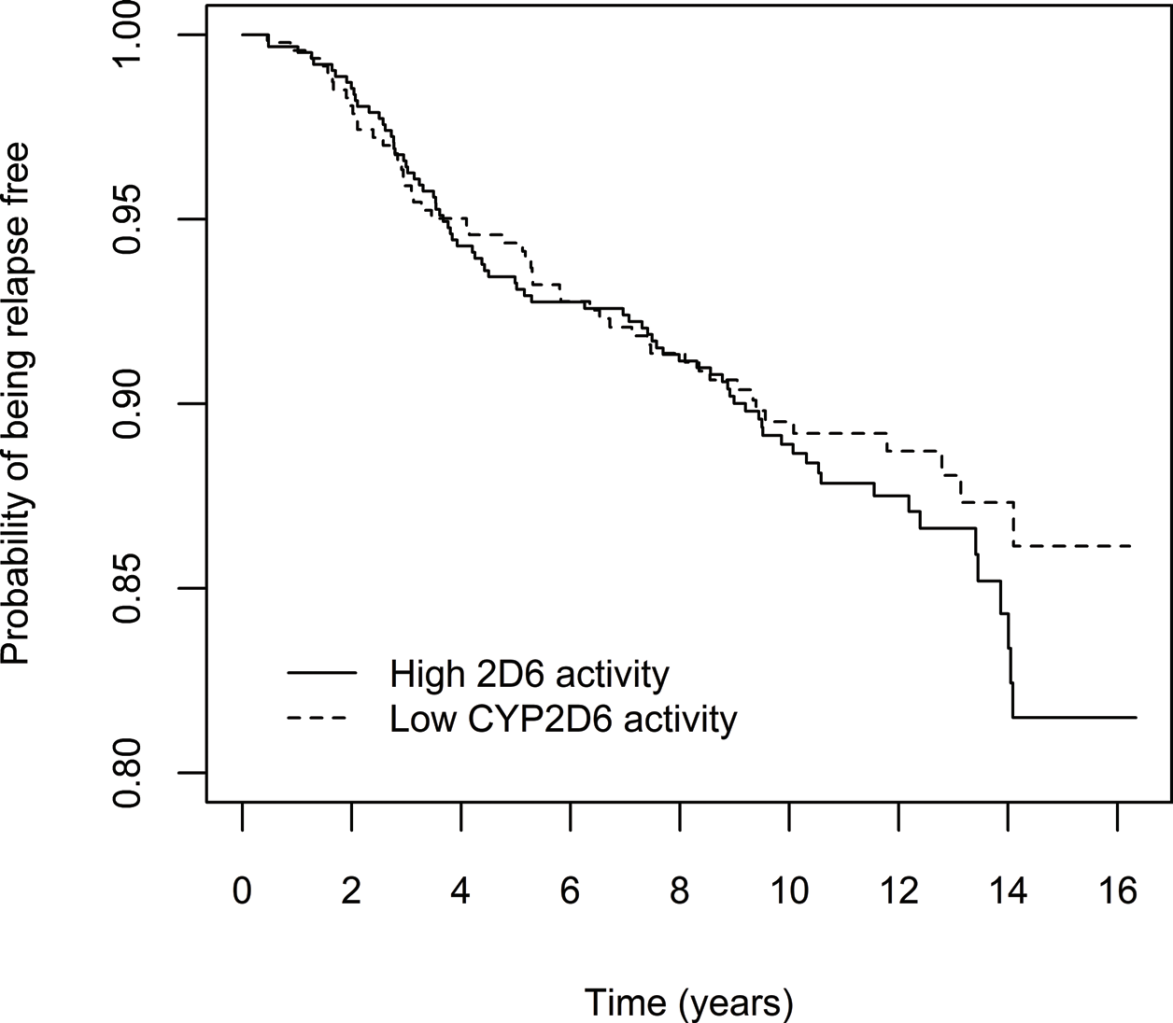
Effect of CYP2D6 activity on breast cancer recurrence, in all patients who initiated adjuvant tamoxifen treatment (n = 1,103). Patients were divided into two groups according to predicted CYP2D6 activity, that is 50% enzyme activity or lower versus higher than 50% activity compared with the ‘normal’ activity encoded by CYP2D6*1/*1. Note that the y-axis is truncated at 0.8.

**Figure 3:**
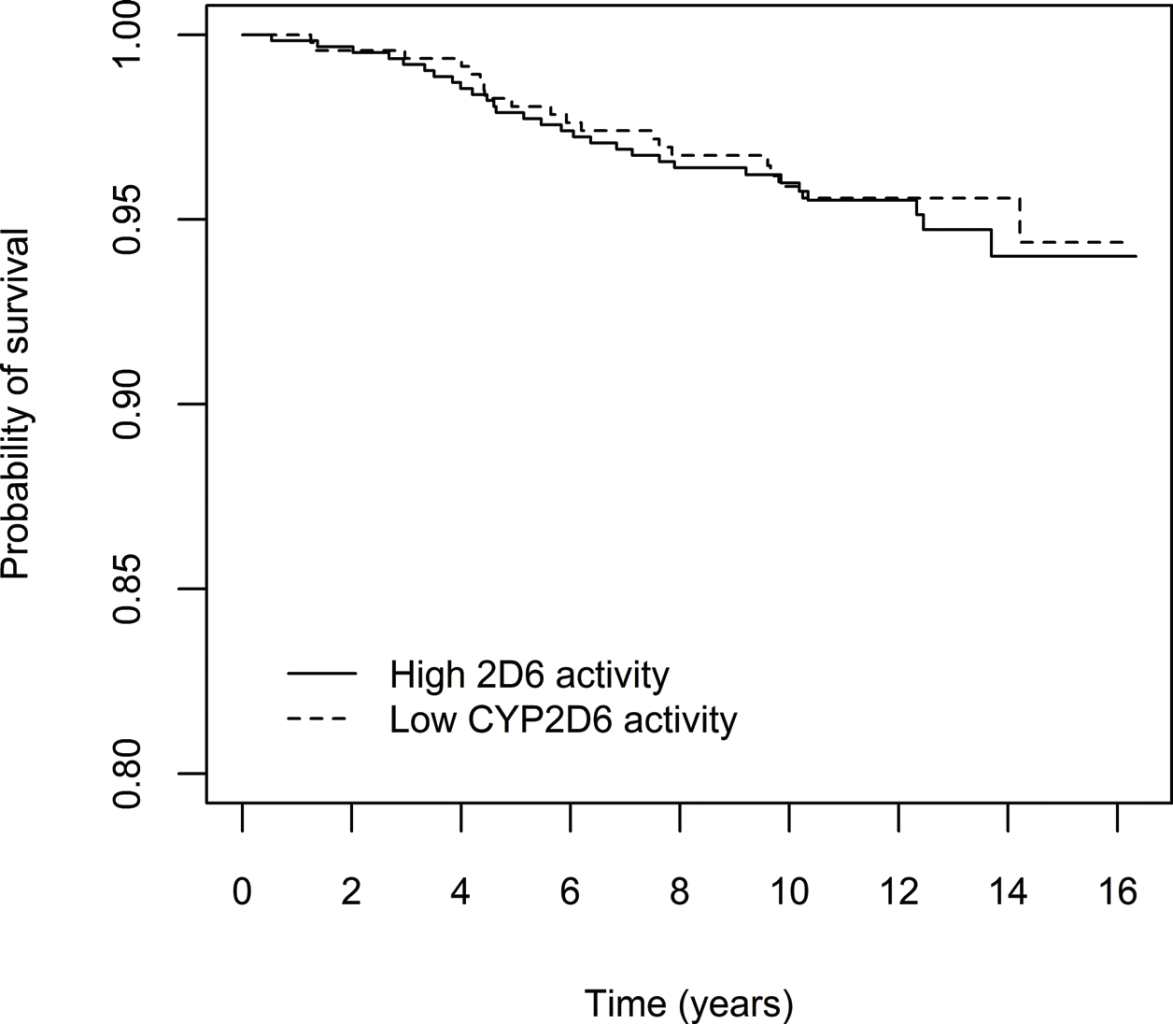
Effect of CYP2D6 activity on breast cancer-specific mortality, in all patients who initiated adjuvant tamoxifen treatment (*n* = 1,103). Patients were divided into two groups according to predicted CYP2D6 activity, that is 50% enzyme activity or lower versus higher than 50% activity compared with the ‘normal’ activity encoded by CYP2D6*1/*1. Note that the y-axis is truncated at 0.8.

**Figure 4:**
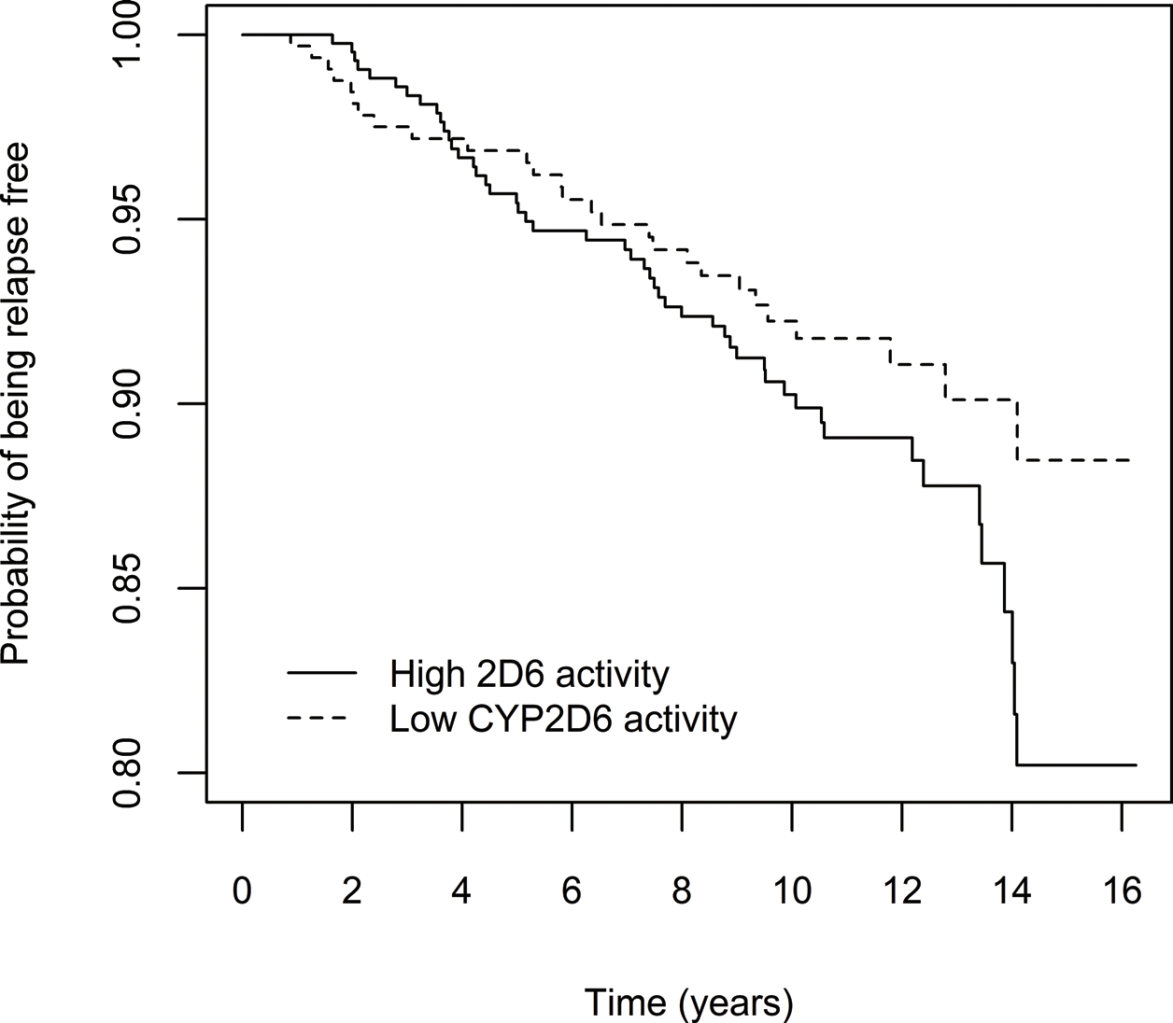
Effect of CYP2D6 activity on breast cancer recurrence, in the subgroup of patients with tamoxifen as their only systemic treatment (*n* = 761). Patients were divided into two groups according to predicted CYP2D6 activity, that is 50% enzyme activity or lower versus higher than 50% activity compared with the ‘normal’ activity encoded by CYP2D6*1/*1. Note that the y-axis is truncated at 0.8.

**Figure 5:**
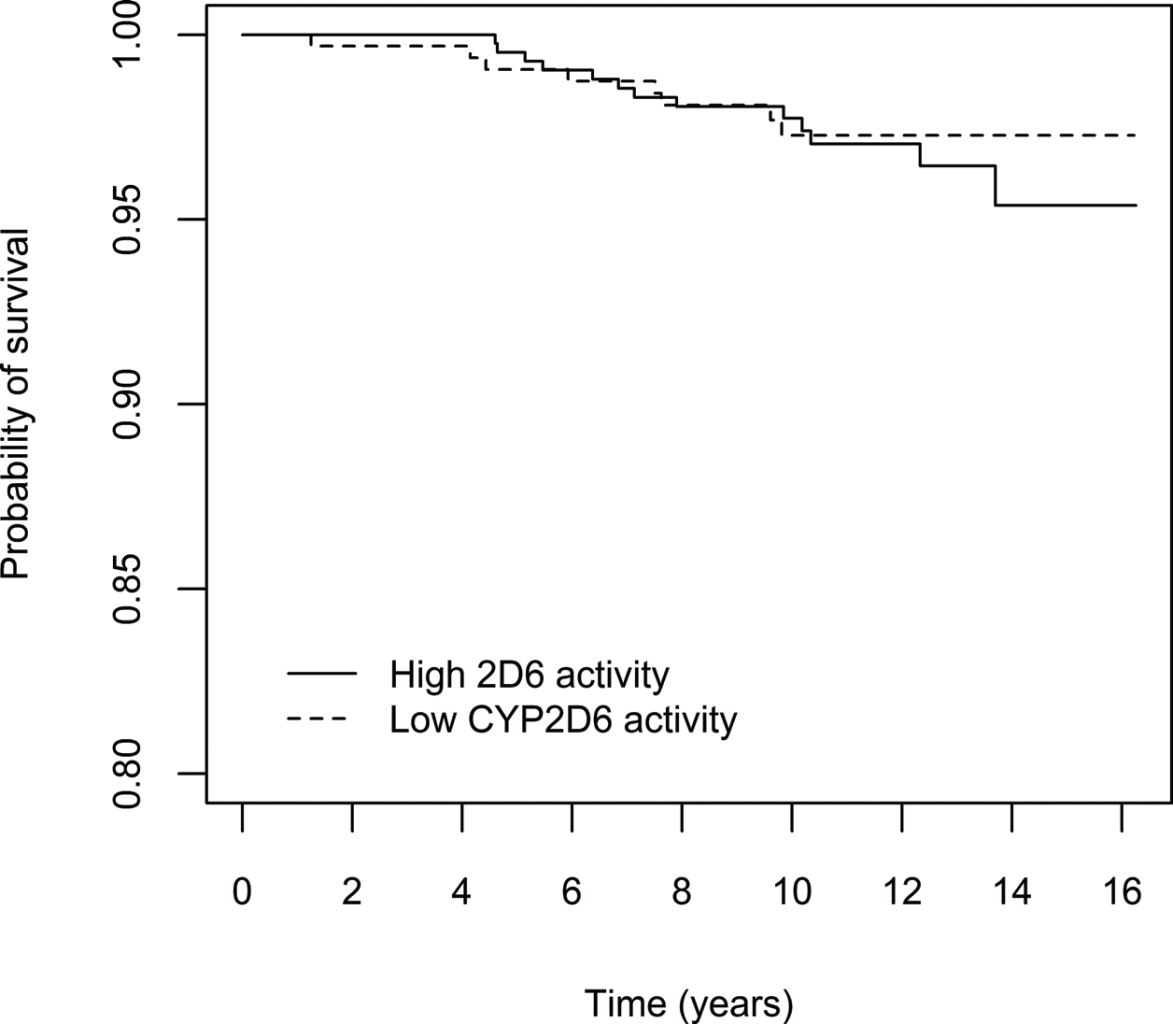
Effect of CYP2D6 activity on breast cancer-specific mortality, in the subgroup of patients with tamoxifen as their only systemic treatment (*n* = 761). Patients were divided into two groups according to predicted CYP2D6 activity, that is 50% enzyme activity or lower versus higher than 50% activity compared with the ‘normal’ activity encoded by CYP2D6*1/*1. Note that the y-axis is truncated at 0.8.

To graphically illustrate the cumulative risk of relapse and breast cancer specific death in the main analysis, (i.e. patients with decreased CYP2D6 activity (50% or less), compared to CYP2D6*1/*1), Kaplan Meier curves in the full cohort are presented in Figures 2 and 3, for the purely tamoxifen treated subgroup in Figures 4 and 5 and for the subgroup of premenopausal women with tamoxifen as their only endocrine treatment in Supplementary Figures 5 and 6.

As shown in Supplementary Figures 1–4, the stratification of patients with tamoxifen monotherapy into low, intermediate, normal and high CYP2D6 activity did not indicate a CYP2D6 gene–dose relationship on clinical outcome.

## Discussion

No association between a reduced CYP2D6 activity and a poorer outcome was found in this population-based cohort of tamoxifen treated pre- and postmenopausal early breast cancer patients, with a long follow-up of 11 years.

The findings from our present investigation indicate that the possible impact of genotype-based prediction of individual CYP2D6 metabolic activity on outcome in tamoxifen treated patients is likely marginal in a modern clinical setting. Our results are in keeping with a prospective study in a similar setting [15]. Another recent study that failed to find a correlation between *CYP2D6* genotype and outcome did observe an association between the plasma level of tamoxifen’s major active metabolite endoxifen, corresponding to a critical threshold of 15 nmol/L to be exceeded for improved prognosis [35]. No such association was however seen when using individual endoxifen concentrations on a continuous scale [35]. It seems possible to speculate that the intra-genotype variability in endoxifen levels, especially among CYP2D6*1/*1-individuals [15, 23, 36–38], might impair the predictive power of genotype on outcome and that therapeutic drug monitoring could rather play a role in dose individualization to ensure that a critical exposure range is reached. In a recent study where a potential threshold of endoxifen at 2–3 ng/mL was indicated, the group of CYP2D6PM was divided for achieving sufficient endoxifen levels for clinical effect [39].

It is possible that a CYP2D6 genotype-dependent effect on clinical outcome may vary in different phases of adjuvant tamoxifen treatment. If this should be the case, considering the pattern of the Kaplan Meier curves in Figures 4, it is possible to speculate whether efficient bio- activation of tamoxifen is especially important during the first years of treatment.

The possible risk of poorer outcome with increasing CYP2D6 activity that was observed in the subgroup analysis of postmenopausal patients is counterintuitive. There is a clear possibility that the counterintuitive finding in this report is caused by unknown confounding factors. Although the signals of a poorer prognosis with increasing CYP2D6 activity should be met with skepticism, further investigations are warranted.

The observed association should not be due to lower adherence to tamoxifen in patients with higher CYP2D6 activity in this material, as adherence was adjusted for in the analysis. Other data has indicated that increasing CYP2D6 activity may be associated with a poorer prognosis, as CYP2D6UM are at risk for prematurely discontinuing therapy, most likely due to pronounced side effects [4]. Importantly, a previous investigation in our study cohort did not indicate that adherence to tamoxifen was poorer in the group of CYP2D6UM compared to those with lower CYP2D6 activity [25]. Earlier studies have rarely accounted for adherence when assessing the role of CYP2D6 genotype in tamoxifen treated patients [5]. In our study from 2013 [9], information on adherence was collected from medical records only, so the true adherence to tamoxifen might be lower.

The relative 5-year survival rate for breast cancer in Sweden has increased from 86% in 1996–2000 to circa 92% in 2022, likely due to earlier diagnosis and improved treatment [40–42]. In this report with patients diagnosed over the period 2006–2014, fewer women, 12%, had a recurrence, only 4% died from breast cancer and OS was 89%, during the follow-up of 11 years. The observed 10-year OS rate in the Stockholm-Gotland region in ER-positive patients diagnosed over the period 2008–2011 was around 80% [22]. Survival in our current study was thus better than average.

In the period when patients in our present cohort were diagnosed, high-risk postmenopausal patients were largely recommended adjuvant treatment with an AI [43]. Most of the patients with tamoxifen as their only endocrine treatment, largely overlapping with the postmenopausal group, in this study were thus generally at lower risk, which likely explains the lower event rate. Moreover, the definition of having a high risk of recurrence in this study was rather wide, which is also reflected by the lower event rate. Improved systemic breast cancer treatment [44–46], not metabolized by CYP2D6, might compensate for reduced tamoxifen activation in patients with poorer CYP2D6 activity.

In the study by Goetz et al. in the ABCSG8-cohort, the negative impact of poor CYP2D6 activity on outcome was seen only when the patients were treated with tamoxifen and not after switching to an AI [8]. We did however not find an association between CYP2D6 activity and outcome in the subgroup with tamoxifen as their only systemic adjuvant treatment either.

## Strengths and limitations

Strengths of this study include the relatively large cohort of prospectively collected early breast cancer patients from a defined geographical region, reflecting progress in standard of care. Detailed clinical data included information on adherence to tamoxifen. *CYP2D6* was extensively genotyped on DNA from blood and follow up was long. Limitations include incomplete prescription data after January 2018. Although we cannot exclude that this could introduce bias, we believe that the missing data is likely of minor importance, as only a minority, 17%, of the patients lacked prescription data, and then only for a fraction of the 5-year period. We accounted for CYP2D6 inhibiting drugs but were not able to discern the duration of concomitant treatment periods. Although *CYP2D6* is the major enzyme involved in the metabolism of tamoxifen, genetic polymorphism in other enzymes such as *CYP2C19,* or nuclear factors that regulate drug metabolism, might also contribute to the variability in tamoxifen bioactivation and response [20, 47]. Another concern is that the low incidence of relapses and breast cancer-related deaths might have rendered the study underpowered for detection of an association between low CYP2D6 activity and clinical outcomes. The point estimates in this study indicate an improved prognosis in CYP2D6 PM. However, the effect was non-significant and from a mechanistic point of view it seems implausible and unlikely to represent a true protective effect of reduced CYP2D6 activity. This notion is further supported by previous studies demonstrating a poorer prognosis in PM [6–8]. Although the non-significant results should not be interpreted as supportive of an *improved* prognosis in CYP2D6 PM, the 95% confidence barely extending below 1 seemingly rules out a substantially *worse* prognosis in these patients. The lower limit of the interval for recurrence, 0.92, corresponds to a risk increase of 19% in CYP2D6 PM compared to NM. It is unlikely (probability < 2.5%) that a true risk increase would be greater than this.

## Conclusion

No association between a reduced CYP2D6 activity and a poorer outcome was found in this cohort of tamoxifen treated early breast cancer patients in a current clinical setting, where adjuvant tamoxifen is mainly used upfront for patients at a lower risk of recurrence. The previously reported association between CYP2D6 activity and outcome in premenopausal tamoxifen treated patients could not be confirmed. A future role of therapeutic drug monitoring to secure sufficient plasma levels and avoid excess exposure associated with intolerability, might still be relevant for patient monitoring.

## Supplementary Material



## Data Availability

The data that support the findings of this study are available from the corresponding author upon reasonable request.
