# Supplementary material for: CYP2D6 genotype and outcome in tamoxifen treated early breast cancer
Source: Acta Oncol. 2025 Jul 2;64:43208. doi: 10.2340/1651-226X.2025.43208 (PMC12239131; doi:10.2340/1651-226X.2025.43208)
Supplement: Supplementary file 1 [file AO-64-43208-s1.pdf]

Supplementary material has been published as submitted. It has not been copyedited, or typeset

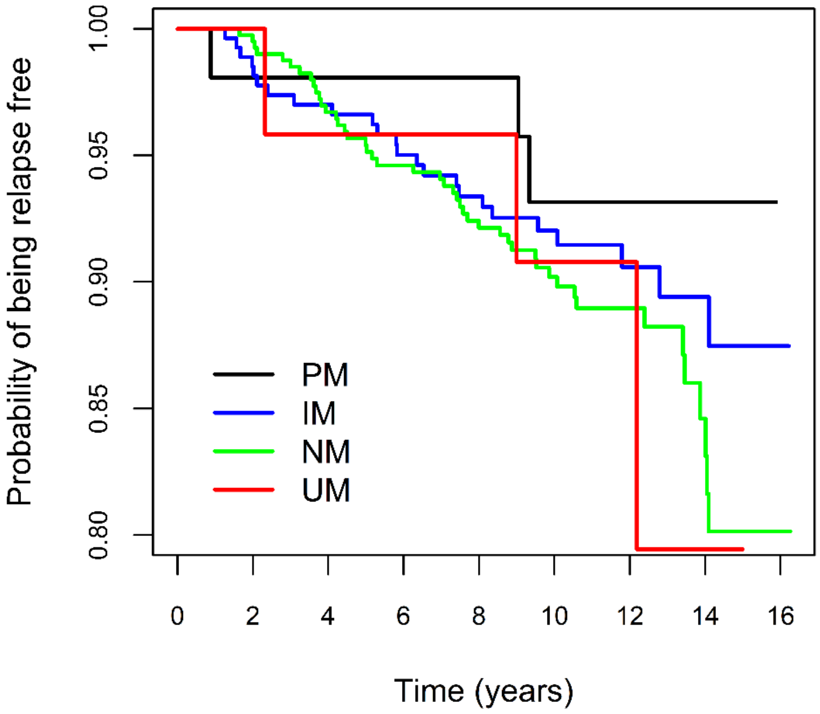

by Acta Oncologica

Suppl Fig 1 Effect of CYP2D6 activity on breast cancer recurrence, in the subgroup of patients with tamoxifen as their only systemic treatment (n = 761). Patients were divided into four groups according to predicted CYP2D6 activity in accordance with recommendations from the Clinical Pharmacogenetics Implementation Consortium and Dutch Pharmacogenetics Working Group [29]. Note that the y-axis is truncated at 0.8.

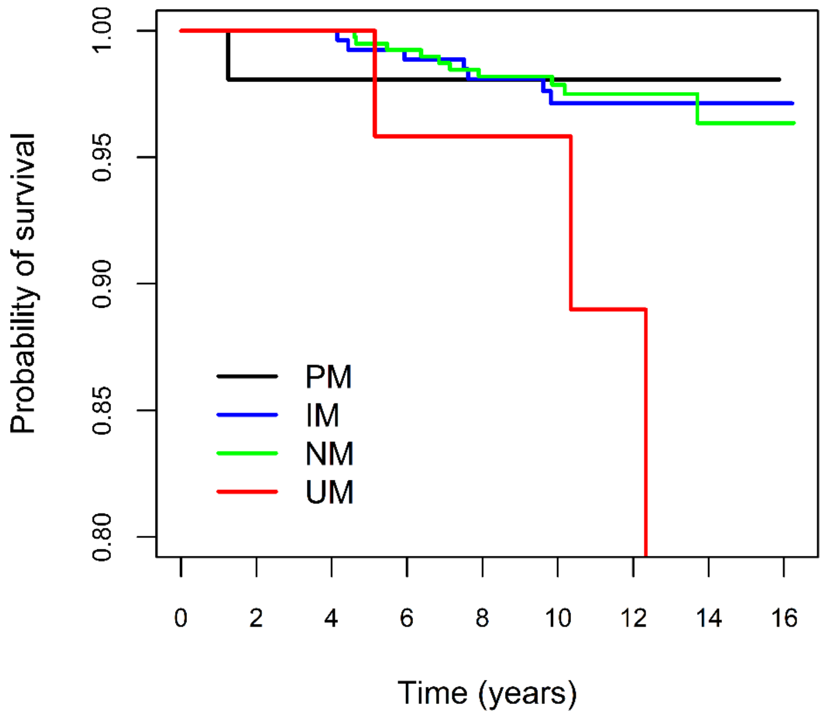

Suppl Fig 2 Effect of CYP2D6 activity on breast cancer mortality, in the subgroup of patients with tamoxifen as their only systemic treatment (n = 761). Patients were divided into four groups according to predicted CYP2D6 activity in accordance with recommendations from the Clinical Pharmacogenetics Implementation Consortium and Dutch Pharmacogenetics Working Group [29]. Note that the y-axis is truncated at 0.8.

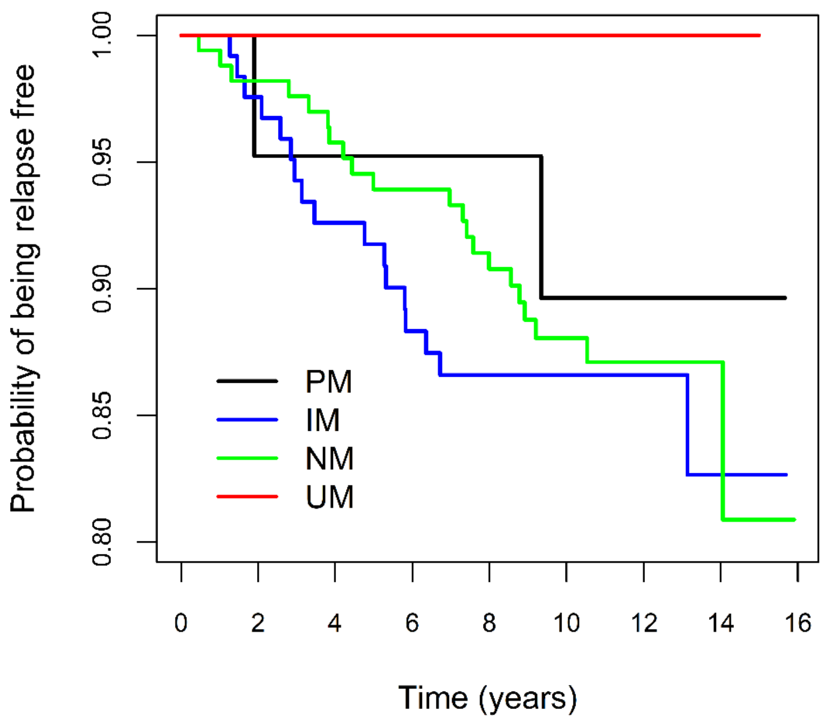

Suppl Fig 3 Effect of CYP2D6 activity on breast cancer recurrence, in the subgroup of premenopausal women with tamoxifen as their only endocrine treatment (n = 325). Patients were divided into four groups according to predicted CYP2D6 activity in accordance with recommendations from the Clinical Pharmacogenetics Implementation Consortium and Dutch Pharmacogenetics Working Group [29]. Note that the y-axis is truncated at 0.8.

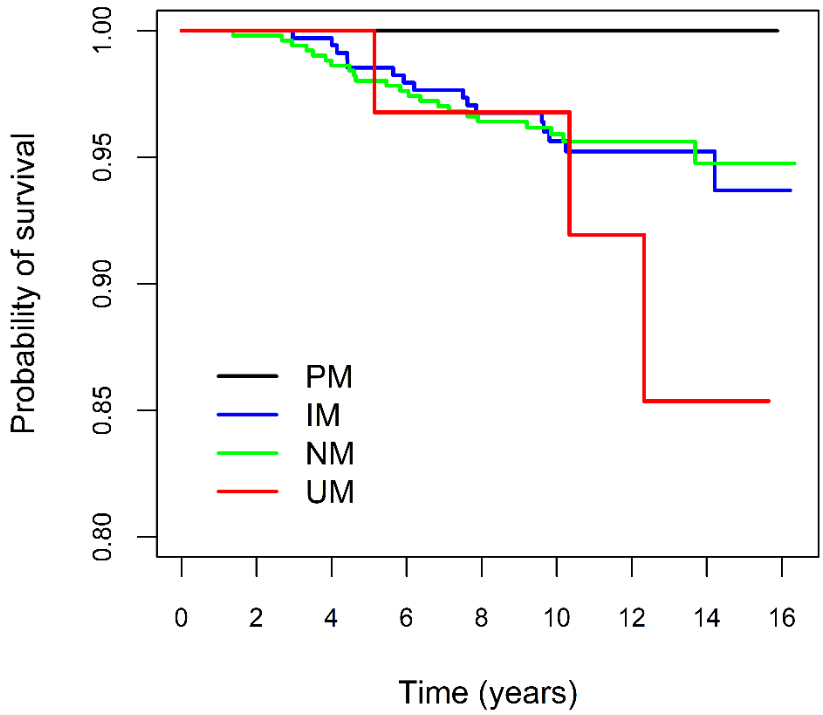

Suppl Fig 4 Effect of CYP2D6 activity on breast cancer mortality, in the subgroup of premenopausal patients with tamoxifen as their only endocrine treatment (n = 325). Patients were divided into four groups according to predicted CYP2D6 activity in accordance with recommendations from the Clinical Pharmacogenetics Implementation Consortium and Dutch Pharmacogenetics Working Group [29]. Note that the y-axis is truncated at 0.8.

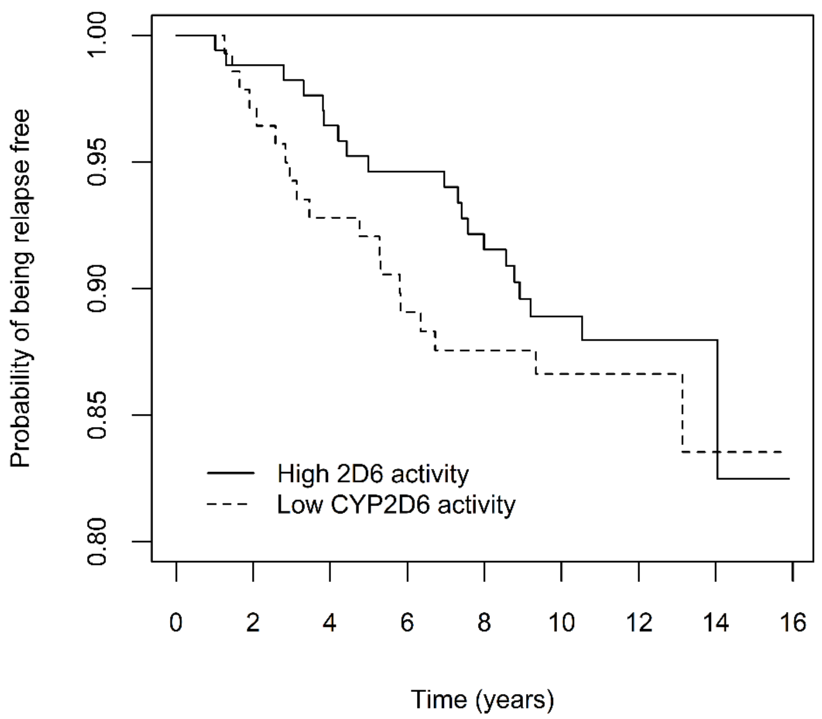

Suppl Fig 5 Effect of CYP2D6 activity on breast cancer recurrence, in the subgroup of premenopausal women with tamoxifen as their only endocrine treatment (n = 325). Patients were divided into two groups according to predicted CYP2D6 activity, i.e., 50 % enzyme activity or lower versus higher than 50 % activity compared with the "normal" activity encoded by *CYP2D6*1/*1*. Note that the y-axis is truncated at 0.8.

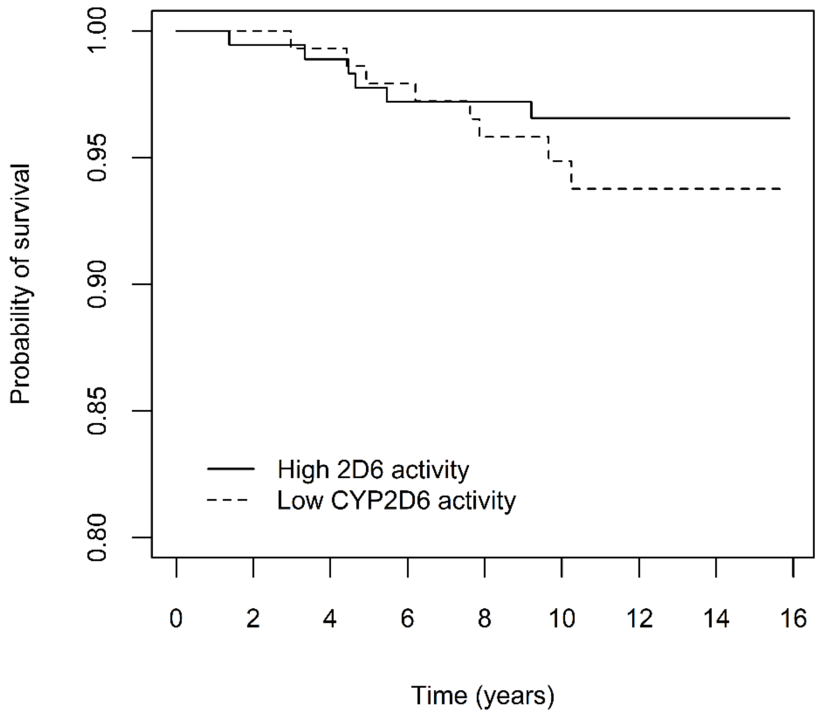

Suppl Fig 6 Effect of CYP2D6 activity on breast cancer-specific mortality, in the subgroup of premenopausal women with tamoxifen as their only endocrine treatment (n = 325). Patients were divided into two groups according to predicted CYP2D6 activity, i.e., 50 % enzyme activity or lower versus higher than 50 % activity compared with the "normal" activity encoded by *CYP2D6*1/*1*. Note that the y-axis is truncated at 0.8.

Supplementary table 1 Association between CYP2D6 activity and relapse

**Patient group**	**Prognostic Covariates**	**Tamoxifen ≥ 1 year (n=1019)**	**CYP2D6 Activity**	**Hazard Ratios, Confidence Intervals / p-value**
**All** (n=1103)		-	Continuous variable	1.18, CI 0.92; 1.52
		Yes		1.12, CI 0.86; 1.47
	Age at cancer diagnosis	-		9.97, CI 0.97; 1.03
	High risk of recurrence	-		1.97, CI 1.32; 2.94
			Low versus high	p=0.4
**Premenopausal** (n=461)		-	Continuous variable	0.99, CI 0.69; 1.42
		Yes		0.98, CI 0.68; 1.42
	Age at cancer diagnosis	-		0.99, CI 0.94; 1.03
	High risk of recurrence	-		1.92, CI 1.00; 3.72
		-	Low versus high	p= 0.8
**Postmenopausal** (n=626)		-	Continuous variable	1.44, CI 0.99; 2.07
		Yes		1.34, CI 0.90; 2.00
	Age at cancer diagnosis	-		1.01, CI 0.97; 1.04
	High risk of recurrence	-		1.81, CI 1.07; 3.08
		-	Low versus high	p= 0.1
**Tamoxifen monotherapy** (n=761)		-	Continuous variable	1.39, CI 0.99; 1.96
	Age at cancer diagnosis	-		1.00, CI 0.97; 1.04
	High risk of recurrence	-		1.87, CI 1.13; 3.08
			Low versus high	p= 0.2
**Tamoxifen monotherapy, premenopausal** (n=176)		-	Continuous variable	1.36, CI 0.71; 2.60
			Low versus high	p=0.2
**Tamoxifen monotherapy, postmenopausal** (n=573)		-	Continuous variable	1.43, CI 0.96; 2.12
			Low versus high	p=0.4
**Tamoxifen only endocrine treatment, all** (n=932)		-	Continuous variable	1.13, CI 0.85; 1.49
	Age at cancer diagnosis	-		1.00, CI 0.97; 1.03
	High risk of recurrence	-		2.03, CI 1.33; 3.10
			High versus low	p= 0.9
**Tamoxifen only endocrine treatment, premenopausal** (n=325)		-	Continuous variable	0.83, CI 0.54; 1.28
	Age at cancer diagnosis	-		1.00, CI 0.94; 1.07
	High risk of recurrence	-		2.08, CI 1.01; 4.27
		-	Low versus high	p= 0.6
**Tamoxifen only endocrine treatment, Postmenopausal** (n=593)		-	Continuous variable	1.44, CI 0.98; 2.09
	Age at cancer diagnosis	-		1.00, CI 0,97; 1.04
	High risk of recurrence	-		1.89, CI 1.09; 3.26
			Low versus high	p= 0.3
**HER2-positive** (n=56, all premenopausal)		-		1.23, CI 0.41; 3.65

The association between CYP2D6 activity and breast cancer relapse was analyzed using multivariable Cox proportional hazard models and logistic regression. Predicted CYP2D6 activity was defined in accordance with recommendations from the Clinical Pharmacogenetics Implementation Consortium and Dutch Pharmacogenetics Working Group [29]. Analyses encoding for both continuous and categorical CYP2D6 activity, i.e. 50 % enzyme activity or lower versus higher than 50 % activity compared with the "normal" activity encoded by *CYP2D6*1/*1*, are presented. Adjustments were in the multivariate model performed for potential confounding variables; age at breast cancer diagnosis, menopausal status, CYP2D6 inhibiting medication at any period during the first five years of follow-up, having a high estimated risk of recurrence and adherence to tamoxifen. Adherence was defined as the proportion of the individual follow-up time (up to five years) that was covered by tamoxifen dispensations. Patients at high risk of recurrence were defined as having positive lymph nodes and /or tumors with high proliferation rate (proliferation index, Ki_{67} > 20 / S phase > 10%) and / or grade III and / or Her2 amplification and / or having received chemotherapy. The associated Hazard Ratios for age at breast cancer diagnosis and having a high estimated risk of recurrence are summarized. Subgroup analyses stratifying patients according to menopausal status, at least one year's initial treatment on tamoxifen, tamoxifen as the only endocrine treatment, HER2-status and tamoxifen monotherapy are presented.

Supplementary table 2 Association between CYP2D6 activity and breast cancer death

Patient group	Prognostic Covariates	Tamoxifen ≥ 1 year (n=1019)	CYP2D6 Activity	Hazard Ratios, Confidence Intervals / p-value
**All** (n=1103)		-	Continuous variable	1.41, CI 0.93; 2.13
		Yes		1.36, CI 0.89; 2.10
	Age at cancer diagnosis	-		0.97, CI 0.97; 1.02
	High risk of recurrence	-		1.97, CI 1.32; 2.94
		-	Low versus high	p=0.8
**Premenopausal** (n=461)		-	Continuous variable	1.14, CI 0.63; 2.05
		Yes		0.99, CI 0.56; 1.77
	Age at cancer diagnosis	-		1.05, CI 0.98; 1.13
	High risk of recurrence	-		7.81, CI 1.81; 33.66
			Low versus high	p= 0.6
**Postmenopausal** (n=626)		-	Continuous variable	1.90, CI 1.02; 3.55
		Yes		2.11, CI 1.08; 4.14
	Age at cancer diagnosis	-		1.07, CI 1.01; 1.11
	High risk of recurrence	-		5.12, CI 2.25; 11.67
			Low versus high	p=0.1
**Tamoxifen monotherapy** (n=761)		-	Continuous variable	1.88, CI 0.98; 3,60
	Age at cancer diagnosis	-		1.09, CI 1.03; 1.15
	High risk of recurrence	-		3.43, CI 1.43; 8.24
			Low versus high	p= 0.6
**Tamoxifen monotherapy, premenopausal** (n=176)	-	-	Continuous variable	1.59, CI 0.21; 12.33
			Low versus high	p= 0.7
**Tamoxifen monotherapy, postmenopausal** (n=573)	-	-	Continuous variable	1.96, CI 0.97; 3.95
			Low versus high	p=0.7
**Tamoxifen only endocrine treatment, all** (n=932)		-	Continuous variable	1.35, CI 0.83; 2.19
	Age at cancer diagnosis	-		1.06, CI 1.02: 1.11
	High risk of recurrence	-		5.27, CI 2.55; 10.92
			Low versus high	p= 0.9
**Tamoxifen only endocrine treatment, premenopausal** (n=325)		-	Continuous variable	0.93, CI 0.43; 2.01
	Age at cancer diagnosis	-		1.10, CI 0.97; 1.26
	High risk of recurrence	-		6.49, CI 1.41; 29.04
		-	Low versus high	p= 0.3
**Tamoxifen only endocrine treatment, postmenopausal** (n=593)		-	Continuous variable	1.82, CI 0.95; 3.48
	Age at cancer diagnosis	-		1.07, CI 1.01; 1.12
	High risk of recurrence			5.29, CI 2.25; 12.47
			Low versus high	p= 0.5
**HER2-positive** (n=56, all premenopausal)		-	Continuous variable	1.11, CI 0.23; 5.42

The association between CYP2D6 activity and breast cancer death was analyzed using multivariable Cox proportional hazard models and logistic regression. Predicted CYP2D6 activity was defined in accordance with recommendations from the Clinical Pharmacogenetics Implementation Consortium and Dutch Pharmacogenetics Working Group [29]. Analyses encoding for both continuos and categorical CYP2D6 activity; i.e. 50 % enzyme activity or lower versus higher than 50 % activity compared with the "normal" activity encoded by *CYP2D6*1/*1*, are presented. Adjustments were in the multivariate model performed for potential confounding variables; age at breast cancer diagnosis, menopausal status, CYP2D6 inhibiting medication at any period during the first five years of follow-up, having a high estimated risk of recurrence and adherence to tamoxifen. Adherence was defined as the proportion of the individual follow-up time (up to five years) that was covered by tamoxifen dispensations. Patients at high risk of recurrence were defined as having positive lymph nodes and /or tumors with high proliferation rate (proliferation index, $Ki_{67} > 20$ / S phase > 10%) and / or grade III and / or Her2 amplification and / or having received chemotherapy. The associated Hazard Ratios for age at breast cancer diagnosis and having a high estimated risk of recurrence are summarized. Subgroup analyses stratifying patients according to menopausal status, at least one year's initial treatment on tamoxifen, tamoxifen as the only endocrine treatment, HER2-status and tamoxifen monotherapy are presented.
